# Force-Controlled Balance Perturbations Associated with Falls in Older People: A Prospective Cohort Study

**DOI:** 10.1371/journal.pone.0070981

**Published:** 2013-08-09

**Authors:** Daina L. Sturnieks, Jasmine Menant, Kim Delbaere, Jos Vanrenterghem, Mark W. Rogers, Richard C. Fitzpatrick, Stephen R. Lord

**Affiliations:** 1 Neuroscience Research Australia and University of New South Wales, Sydney, New South Wales, Australia; 2 School of Sport and Exercise Sciences, Liverpool John Moores University, Liverpool, United Kingdom; 3 Department of Physical Therapy and Rehabilitation Science, University of Maryland School of Medicine, Baltimore, Maryland, United States of America; UCSD School of Medicine, United States of America

## Abstract

Balance recovery from an unpredictable postural perturbation can be a challenging task for many older people and poor recovery could contribute to their risk of falls. This study examined associations between responses to unpredictable perturbations and fall risk in older people. 242 older adults (80.0±4.4 years) underwent assessments of stepping responses to multi-directional force-controlled waist-pull perturbations. Participants returned monthly falls calendars for the subsequent 12 months. Future falls were associated with lower force thresholds for stepping in the posterior and lateral but not anterior directions. Those with lower posterior force thresholds for stepping were 68% more likely to fall at home than those with higher force thresholds for stepping. These results suggest that amount of force that can be withstood following an unpredictable balance perturbation predicts future falls in community-dwelling older adults. Perturbations in the posterior direction best discriminated between future fallers and non-fallers.

## Introduction

Responding to an unpredictable balance perturbation is a challenging task. With no prior information, sensory information that describes the nature of the perturbation must be received, assessed quickly and accurately, to determine and execute the appropriate motor response to avoid falling. Depending on the size of the perturbation and the individual’s capacity to respond appropriately, it may be possible to recover balance using a feet-in-place postural sway response. However, for larger perturbations that threaten to move the body centre of mass beyond the base of support limits, it is (or is perceived to be [1], [2]) necessary to increase or re-position the base of support, for example, by taking a step.

Previous studies, employing various experimental procedures to perturb balance, have identified the importance of stepping and its critical factors for effective balance control [3]–[5]. Inappropriate step responses are more prevalent in older compared with younger people; while young people respond by taking a single step, older people take multiple shorter steps [6]–[8] and are more likely to contact the contralateral limb, leading to further instability [8]–[10]. Impaired stepping is even more common in older people at risk of falls and those with balance impairments. For example, we have previously found that older people with high physiological risk of falling are able to withstand less forceful waist pulls compared with those with low fall risk [11]. “Laboratory” falls triggered by induced trips and platform or waist-pull perturbations are associated with the degree of trunk flexion, the size of the recovery first step, lower limb moments and rate of moment generation [12], [13]. Thus, it seems that the inability to recover balance following an unpredictable perturbation may be a recognizable cause of falls in older people [7].

Few prospective studies have investigated the capacity of unpredictable perturbation response measures to predict falls among community-living older people [14]–[16]. In one study of 64 older adults, Maki and colleagues reported trends for an increased risk of falls following perturbation of the support surface [16]. People who responded by taking multiple steps were more likely to experience a fall to the side (*p* = 0.055), while people who responded by taking more laterally directed steps were more likely to fall forward or backward (*p* = 0.067). Another study employing unpredictable surface perturbations found the ability to control postural sway was moderately accurate in predicting future fallers in 100 older adults [15]. Hilliard et al investigated unpredictable lateral waist-pull perturbations in 50 older people and found that those who responded to all trials with multiple steps were six times more likely to fall in the subsequent 12 months than those who did not always use multiple steps [14]. These studies examined responses that were sub- and supra-threshold for stepping. No studies have examined an individual’s ability to withstand different levels of force perturbation or the magnitude threshold that induces stepping with respect to fall risk.

Clearly, further research is required to understand stepping responses, including the assessment of whether a reduced capacity to withstand perturbation forces can identify older people at risk of falls. This study investigated whether force thresholds for stepping, induced by unpredictable waist-pull balance perturbations [11], were predictive of fallers and at-home fallers living in the community. We hypothesised that fallers would have lower force thresholds for stepping, take more poorly directed steps, take shorter steps and require multiple steps, make more cross-steps, and exhibit slower step initiation times than non-fallers.

## Methods

### Participants

Two hundred and forty-two community-dwelling older adults (132 men, 110 women with mean age 80.0 years, SD = 4.4) participated in this study. These participants were recruited from a larger longitudinal study of cognitive function and ageing (Sydney Memory & Ageing Study) conducted in eastern Sydney, Australia [17]. Inclusion criteria included living independently in the community and being able to walk 400 m without assistance. Study exclusions included minimal English language skills, neurological, musculoskeletal or cardiovascular impairment that would prevent the undertaking of assessments, and Mini-Mental State Examination score of <24 [18]. The study was approved by the University of New South Wales Human Research Ethics Committee and all participants provided informed written consent prior to participation.

### Demographic, Health and Falls Efficacy Measures

Participants completed structured interviews and questionnaires regarding demographics, general health (12-item World Health Organization Disability Assessment Schedule - WHODAS II [19]), major medical conditions, medication use and fear of falling using the Falls Efficacy Scale - International (FES-I). The FES-I is a widely used assessment of concern about falls when carrying out activities and ranges from 16 (low fear) to 64 (high fear) [20].

### Waist-pull Balance Perturbations

**Figure 1:**
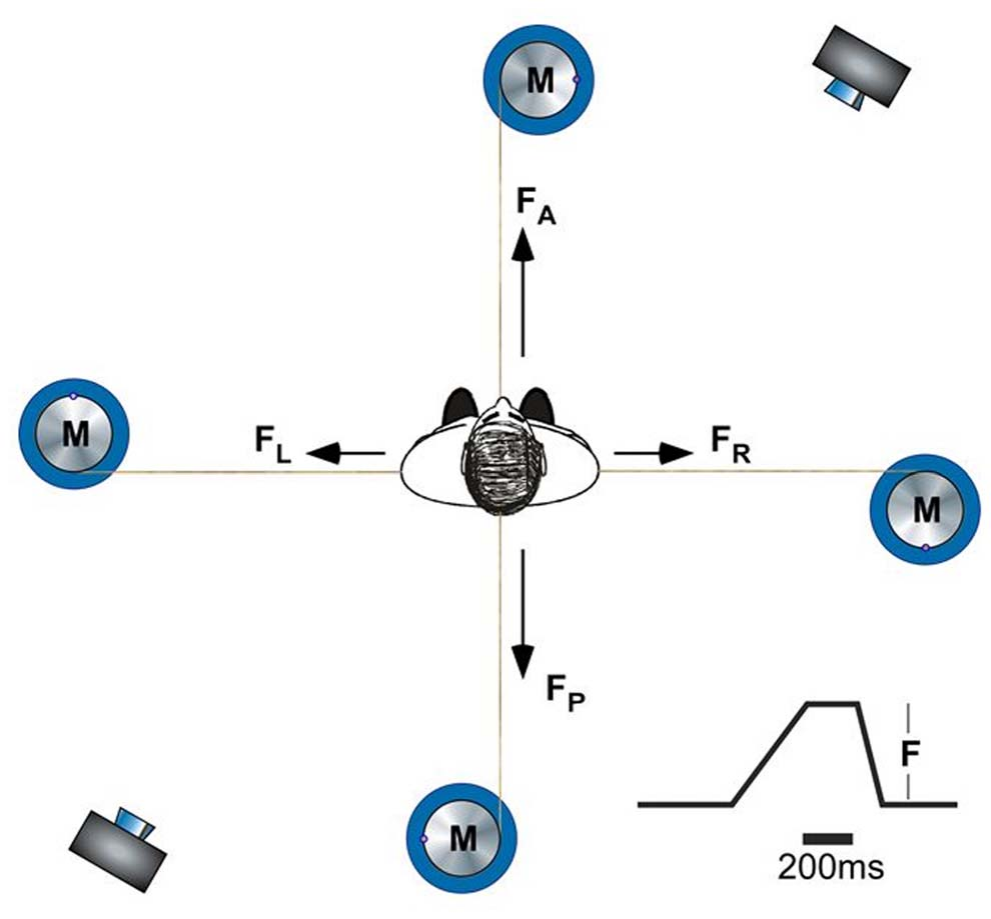
Waist-pull balance perturbation setup, showing camera and motor (M) positions, force (F) directions (A = anterior; P = posterior, R = right lateral, L = left lateral) and perturbation force profile.

To ensure safety, participants wore a harness that did not restrict stepping movements. Markers were placed on the calcaneus, first and fifth metatarsophalangeal joints of the feet and were acquired at 100 Hz using two CODA cx1scanner units (Codamotion, Charnwood Dynamics Ltd., Rothley, UK). Participants wore comfortable footwear and stood relaxed, with a hip-width stance, connected to a motor via cables extending from a belt fixed firmly around the pelvis (Figure 1). At a random time interval, the motor applied a constant force for 0.5 s in different (forward, backward, left, right) directions. To minimise overshoot of the perturbation force, the profile of the stimulus was 300 ms ramp up to target force, 200 ms hold and 100 ms ramp off. Participants were told to try to maintain their balance and only step if necessary to prevent falling. Perturbation directions were block randomised by anterior/posterior and left/right directions. An estimated perturbation threshold (E) was calculated with an equation based on the participant’s body weight (BW) [11]: for antero-posterior perturbations, E = (0.422*BW)+35.0; and for lateral perturbations, E = (0.1448*BW)-23.1. Perturbation forces were randomly presented at E−5N, E−10N, E+5N, E+10N. If a step was not induced during this first block of forces, a block of increased forces (+15N, +20N) was introduced and randomly presented. This method was repeated until a step was induced. Participants received 22 perturbations, on average, including one practice trial in each direction. The protocol took approximately 10 min, excluding marker placement.

### Falls

Falls were defined as unintentionally coming to the ground or some lower level and other than as a consequence of sustaining a violent blow, loss of consciousness, sudden onset of paralysis as in stroke or an epileptic seizure [21]. Fall frequency during a one-year prospective period was monitored with monthly falls diaries and follow-up telephone calls as required [22]. For our analyses, fallers were defined as those who reported one or more falls during the 12 month follow-up period. The sub-group of fallers who fell at home were also contrasted with the remainder of participants as it has been found that indoor falls are associated with reduced physical functioning [26] and that indoor fallers suffer high rates of future immobility [23] and fall-related mortality [24].

### Data Analyses

The lowest level of delivered perturbation force (N) at which a participant took a step to maintain balance was recorded as the force threshold for stepping. These thresholds were determined for anterior, posterior and lateral pulls. Using Visual3D (C-Motion, Germantown, MD, USA), foot marker coordinates were traced to calculate the following kinematic variables for the threshold perturbation trial: step initiation time (s, from perturbation onset to toe off), first step velocity (m/s), first step length (m), first step direction (angle deviation from the line of pull) and number of steps. Due to camera positions, kinematic data were only available for anterior and posterior perturbation trials only. The stepping strategy for lateral pulls was observed by the researcher and categorised as either a side-step or a crossover step.

### Statistical Analyses

FES-I and step direction data had right skew distribution and were therefore log-transformed for parametric tests (tables in results present raw, non-transformed data). All statistical analyses were performed using IBM SPSS Statistics 20, with the significance level set at *p*<0.05. T-tests for continuously scaled variables and Chi square tests for contingency tables were used to compare baseline data between the faller groups. Between-group comparisons in continuous variables (force thresholds, step initiation time, velocity, length and direction) were assessed using ANCOVA, controlling for height and weight. To investigate potential threshold effects, stepping performance was dichotomized at the median (for variables meeting the criteria of *p*<0.1 from these ANCOVAs) and used to calculate Relative Risks (RR) and 95% Confidence Intervals (CI) for faller status.

## Results

One hundred and six participants (44%) reported at least one fall in the 12-month follow-up period, of whom 54 (22%) reported a fall at home. All at-home falls occurred during usual activities of daily living. Demographic and health characteristics for the faller groups are presented in Table 1. Overall, the participants scored high on the Mini-Mental State Examination and reported low to moderate concern regarding falls as indicated by their FES-I scores. The majority of participants (98%) rated their health as being good to very good on the WHODAS II. The fallers and at-home fallers were similar to their respective non-faller comparison group for age, height, weight, gender, overall health and cognitive function.

**Table 1:** Demographic, health and falls characteristics for the whole sample, non-fallers versus fallers and people who had at least one fall at home versus people who had no falls or no falls at home.

	Total	Non-fallers	Fallers	Non at-home fallers	At-home fallers
	(n = 242)	(n = 136)	(n = 106)	(n = 188)	(n = 54)
Sex (female)	110 (46%)	61 (45%)	49 (46%)	90 (48%)	20 (37%)
Age (years)	80.0 (4.4)	80.2 (4.5)	79.8 (4.3)	79.9 (4.3)	80.3 (4.5)
Height (cm)	164.3 (9.1)	163.5 (8.9)	165.2 (9.3)	163.9 (8.9)	165.7 (9.9)
Weight (kg)	71.4 (13.1)	71.0 (13.2)	71.8 (13.1)	71.0 (13.1)	72.6 (13.2)
MMSE score a	29.1 (1.3)	29.2 (1.2)	29.1 (1.4)	29.1 (1.3)	29.1 (1.3)
WHODAS II b	18.4 (6.3)	18.3 (6.6)	18.5 (5.8)	18.3 (6.3)	18.8 (6.1)
FES-I score c	21.8 (5.3)	21.0 (4.6)	22.7 (6.0) *	21.5 (4.9)	22.7 (6.5)
≥2 falls in past year	35 (15%)	14 (10%)	21 (20%) *	22 (12%)	13 (24%) ?
>4 medications	137 (57%)	76 (56%)	61 (58%)	100 (54%)	37 (69%) ?

Data are presented as mean (SD) or number (%).

a Mini Mental State Examination (score range 0–30) – adjusted for age, years of education and non-English speaking background [18].

b 12-item World Health Organization Disability Assessment Schedule II (score range 0–36) [19].

c Falls Efficacy Scale – International (score range 16–64) [20].

* Significantly different to fallers (p<0.05).

?Significantly different to non at-home fallers (p<0.05).

### Perturbation Responses: Faller and Non-faller Comparisons

Force thresholds for stepping and step characteristics for the faller groups are presented in Table 2. These results show that fallers had significantly reduced posterior stepping force thresholds (F_3,239_ = 4.446, *p* = 0.036), but similar anterior and lateral force thresholds for stepping compared with non-fallers (F_3,239_ = 0.401, *p* = 0.527; F_3,239_ = 1.531, *p* = 0.217, respectively). Step initiation times, step velocity, step length and step direction in response to both anterior and posterior threshold perturbations were similar in fallers and non-fallers. Similar proportions of fallers and non-fallers took multiple steps in response to their three directional threshold perturbations and there was no difference in the proportions of fallers and non-fallers that used the cross-step strategy in response to their threshold lateral perturbation. There was a trend suggesting that participants with lower posterior force thresholds for stepping (median cut-off) were more likely to fall than participants with higher thresholds (RR = 1.29, 95%CI = 0.96–1.73, *p* = 0.089).

**Table 2:** Force thresholds for stepping, step initiation time, initial step length and stepping strategy for the total sample, fallers and non-fallers, as well as at-home fallers and non at-home faller subgroups.

	Direction	Total	Non-fallers	Fallers	Non at-home fallers	At-home fallers
		n = 242	n = 136	n = 106	n = 188	n = 54
Force threshold (N)	anterior	50.3 (14.0)	50.9 (13.9)	49.8 (12.8)	50.8 (13.3)	48.9 (14.0)
	posterior	45.6 (13.2)	46.6 (13.0)	44.2 (13.0)*	46.5 (12.8)	42.8 (13.5) ?
	lateral	70.5 (21.1)	72.6 (21.6)	70.0 (21.8)	72.7 (20.9)	67.9 (22.7) ?
Step initiation time (s)	anterior	0.83 (0.30)	0.83 (0.33)	0.83 (0.26)	0.82(0.31)	0.87 (0.25)
	posterior	0.75 (0.25)	0.74 (0.21)	0.76 (0.30)	0.72 (0.21)	0.84 (0.35) ?
Step velocity (m/s)	anterior	1.02 (0.43)	1.05 (0.44)	0.98 (0.42)	1.02 (0.44)	1.00 (0.40)
	posterior	0.87 (0.40)	0.88 (0.39)	0.86 (0.41)	0.87 (0.38)	0.88 (0.47)
Step length (m)	anterior	0.20 (0.12)	0.20 (0.12)	0.19 (0.12)	0.19 (0.12)	0.21 (0.13)
	posterior	0.17 (0.10)	0.17 (0.10)	0.17 (0.10)	0.17 (0.10)	0.18 (0.12)
Step direction (deg)	anterior	9.9 (8.7)	10.0 (8.0)	9.6 (9.8)	9.9 (8.3)	9.8 (10.5)
	posterior	8.9 (7.6)	9.5 (7.8)	8.3 (7.2)	9.2 (8.0)	8.2 (5.4)
Multiple steps (%)	anterior	52 (22)	27 (21)	25 (25)	39 (22)	15 (25)
	posterior	86 (36)	46 (37)	40 (39)	66 (38)	20 (39)
	lateral	110 (79)	62 (60)	48 (55)	84 (56)	26 (62)
Cross-step strategy (%)	lateral	131 (69)	72 (71)	59 (68)	104 (71)	27 (64)

Data presented as Mean (SD), except multiple steps and stepping strategy which are presented as number (%).

* Significantly different to fallers (p<0.05) after controlling for height and weight.

?Significantly different to non at-home fallers (p<0.05) after controlling for height and weight.

### Perturbation Responses: At-home Faller and Non at-home Faller Comparisons

At home fallers had significantly reduced posterior and lateral stepping force thresholds (F_3,239_ = 5.890, *p* = 0.016; F_3,239_ = 4.975, *p* = 0.027, respectively) and similar anterior stepping force thresholds to those who did not fall at home (F_3,239_ = 1.249, *p* = 0.265). At-home fallers had significantly slower step initiation times in response to their posterior threshold perturbation (F_3,239_ = 7.922, *p* = 0.005). Initial step velocity, step length and step direction in response to both anterior and posterior threshold perturbations were similar in at-home fallers and non at-home fallers. Similar proportions of at-home fallers and non at-home fallers took multiple steps in response to their three directional threshold perturbations and there was no difference in the proportions of at-home fallers and non at-home fallers that used the cross-stepstrategy in response to their threshold lateral perturbation. Participants with lower posterior force thresholds for stepping (median cut-off) were 68% more likely to fall at home than participants with higher thresholds (RR = 1.68, 95%CI = 1.01–2.80). There was no threshold effect regarding lateral force thresholds for stepping (RR = 1.01, 95%CI = 0.62–1.65). Participants with slower posterior step initiation times (median cut-off) were almost twice as likely to fall at home than participants with faster step initiation times (RR = 1.89, 95%CI = 1.27–3.19).

## Discussion

A large proportion (44%) of the sample reported one or more falls in the 12-month follow-up period. These falls included those that occurred during normal activities of daily living, as well as resulting from sports and extraordinary activities. This high rate of falls, relative to previous reports in community-dwelling older adults [25], [26], may be due to the older average age of the sample or increased diligence associated with long-term involvement in a falls research study. A smaller proportion of participants reported at-home falls during normal activities of daily living (22%), which may be more strongly associated with reduced physical functioning [27].

Force thresholds for stepping in the posterior direction were significantly associated with future falls in both the faller group as a whole and the sub-group who fell at home. A low (below median) posterior force threshold for stepping represented a 68% increased risk of falls at home. In addition, participants with slower step initiation times in response to posterior pulls were almost twice as likely to fall at home. Posterior balance recovery induced by either waist pulls as used here or by forward platform perturbations [28] are particularly challenging as the centre of mass-to-base of support border is relatively short in this direction and requires a quick compensatory step to prevent falling. The ankle plantarflexor muscle moment that supports the body against gravity during normal standing is immediately available to eccentrically resist a forward fall. This provides a relatively easier task for the postural control system, compared to a posterior perturbation, for which the knee and anterior tibial muscles are important for overcoming the flow of perturbation energy to the upper body [28]. The finding that the more challenging posterior perturbations discriminated best between faller and non-faller groups lends support to the posterior-directed retropulsion test as part of a fall risk assessment. This test evaluates an individual’s ability to recover from a backward pull on the shoulders and has been a very useful clinical test of balance control in people with Parkinson’s disease. Future studies should explore its validity as a fall risk screen in both clinical groups and healthy older adults.

It has been suggested that older people might be particularly vulnerable to lateral instability [15], [29], with studies showing older adults require more steps and arm reactions, have frequent collisions between the limbs and increased trunk motion in response to lateral balance perturbations [8]–[10]. In one prospective study, Maki et al [15] found fallers had significantly larger amounts of lateral sway following unpredictable platform perturbations. In the present study, lateral thresholds for stepping were significantly reduced in the at-home fallers, but not the faller group as a whole. Furthermore, the need to take multiple steps in response to lateral perturbations is a strong indicator of fall risk [8], [14]. In a prospective study of 51 older people, Hilliard et al [14] found participants who used a multiple step response to regain balance in response to a lateral waist pull perturbation were more than six times more likely to fall in the subsequent year, compared to participants who did not always require a multiple step response. Finally, it has also been recently reported that in a study of 75 older people, older fallers have particular difficulty with lateral perturbations when exposed to 12 randomly applied waist-pull perturbation directions [8].

In the present study, fallers and non-fallers had similar rates of multiple stepping and used a similar proportion of cross and side-stepping strategies. This is likely due to a different study design, being that our study only examined stepping responses at the threshold step, where Hilliard and colleagues examined responses from 10 trials employing a supra-threshold perturbation that would be more destabilising and require a greater energy and power output to counter. These supra-threshold perturbations were also position controlled, meaning that the pelvis is moved a given distance at a given velocity regardless of the individual’s response. In the current study, delivering force-controlled perturbations, it was possible for participants to mount an opposing force to control their centre of mass position, which might have prolonged the time before a step was taken and therefore altered the step characteristics. Nonetheless, it is possible that examining multiple step behaviours in all perturbation trials would have been more indicative of an older persons fall risk.

Similar to findings from Hilliard and co-authors [14], the proportion of cross- and side-steppers in this study were not different between faller and non-faller groups. The majority of participants chose the cross-step strategy, which is consistent with a previous study of stepping involving older people [10]. The increased time spent on one leg, the more convoluted step path and risk of inter-limb collision makes the cross-step a more hazardous choice, but is likely forced by the older adult’s inability to unload the limb ipsilateral to the perturbation direction, in order to withdraw this foot for a side-step. Although taking a side-step is considered to be the safer stepping strategy, it is probable that few older adults have the muscular strength and central resources necessary for effectively performing this manoeuvre. A limitation of our study was our side-step classification. Previous studies have differentiated between stepping with the leg that was loaded as a result of the perturbation-induced body motion and a neuromechanically easier side-stepping strategy that involves an initial small medial side-step with the contralateral (unloaded) leg. As we did not differentiate between these two strategies, it is likely that some of our participants categorised as side-steppers used this simpler latter strategy [8], [9].

To determine force thresholds for stepping, participants were asked to try to keep their feet in place and only step to avoid falling. It is likely, however, that some participants chose to step before it was (mechanically) necessary to avoid falling. Pai and colleagues found older people often take a step well-before the support limits are reached, especially those who have experienced a fall in the past [1]. It is possible that the force thresholds reflect a decision to step, based on prior experience, fear and/or an inappropriate notion of one’s own capacity, rather than an absolute need. The degree of this ‘cautious’ stepping behaviour is likely to influence the step characteristics examined. Also, people may step well below threshold when left to respond naturally, suggesting that the responses seen in the current study (with instructions “try not to step”) may not accurately reflect all responses in daily life [30]. However, regardless of any disparity between the actual or perceived need to step or whether the experimental set-up resembles daily life behaviour, this study has shown that the amount of force that is withstood following the unpredictable balance perturbation employed, particularly in the posterior direction, was predictive of falls in community-dwelling older adults.

Finally, initial studies have shown that younger and older adults are able to learn to resist loss of balance with repeated exposure to perturbations. A significant reduction in the incidence of fall and balance loss was achieved within in a single session of 24 backward perturbation (slip) trials, with significant retention of this ability at 6 months [31]. The number of steps required to maintain balance in response to waist pulls has also been shown to decrease over 60 repeated trials [32]. Furthermore, a 6-week perturbation-based training intervention led to reductions in frequency of multi-step reactions, foot collisions and handrail contact time, compared with a control intervention of flexibility and relaxation training [33]. These encouraging findings suggest training the perturbation response might be an effective fall prevention intervention.
